# Overview of CAPICE—Childhood and Adolescence Psychopathology: unravelling the complex etiology by a large Interdisciplinary Collaboration in Europe—an EU Marie Skłodowska-Curie International Training Network

**DOI:** 10.1007/s00787-020-01713-2

**Published:** 2021-01-20

**Authors:** Hema Sekhar Reddy Rajula, Mirko Manchia, Kratika Agarwal, Wonuola A. Akingbuwa, Andrea G. Allegrini, Elizabeth Diemer, Sabrina Doering, Elis Haan, Eshim S. Jami, Ville Karhunen, Marica Leone, Laura Schellhas, Ashley Thompson, Stéphanie M. van den Berg, Sarah E. Bergen, Ralf Kuja-Halkola, Anke R. Hammerschlag, Marjo Riitta Järvelin, Amy Leval, Paul Lichtenstein, Sebastian Lundstrom, Matteo Mauri, Marcus R. Munafò, David Myers, Robert Plomin, Kaili Rimfeld, Henning Tiemeier, Eivind Ystrom, Vassilios Fanos, Meike Bartels, Christel M. Middeldorp

**Affiliations:** 1 Neonatal Intensive Care Unit, Department of Surgical Sciences, AOU and University of Cagliari, Cagliari, Italy; 2 Section of Psychiatry, Department of Medical Science and Public Health, University of Cagliari, Cagliari, Italy; 3 Department of Pharmacology, Dalhousie University, Halifax, NS Canada; 4 Department of Learning, Data Analytics and Technology, University of Twente, Enschede, The Netherlands; 5 Department of Biological Psychology, Vrije Universiteit Amsterdam, Amsterdam, The Netherlands; 6 Social, Genetic and Developmental Psychiatry Centre, Institute of Psychiatry, Psychology and Neuroscience, King’s College London, London, UK; 7 Child and Adolescent Psychiatry, Erasmus University Medical Centre, Rotterdam, The Netherlands; 8 Centre for Ethics, Law and Mental Health (CELAM), Gillberg Neuropsychiatry Centre, University of Gothenburg, Gothenburg, Sweden; 9 MRC Integrative Epidemiology Unit, University of Bristol, Bristol, UK; 10 School of Psychological Science, University of Bristol, Bristol, UK; 11 Department of Epidemiology and Biostatistics, MRC-PHE Centre for Environment and Health, School of Public Health, Imperial College London, London, UK; 12 Janssen Pharmaceutical, Global Commercial Strategy Organization, Stockholm, Sweden; 13 Department of Medical Epidemiology and Biostatistics, Karolinska Institutet, Stockholm, Sweden; 14 Amsterdam Public Health Research Institute, Amsterdam, The Netherlands; 15 Faculty of Medicine, Center for Life Course Health Research, University of Oulu, Oulun yliopisto, Oulu, Finland; 16 Biocenter Oulu, University of Oulu, Oulu, Finland; 17 Unit of Primary Health Care, Oulu University Hospital, Oulu, Finland; 18 Department of Life Sciences, College of Health and Life Sciences, Brunel University , London, UK; 19 University of Cagliari, Cagliari, Italy; 20 PROMENTA Research Center, Department of Psychology, University of Oslo, Oslo, Norway; 21 Norwegian Institute of Public Health, Oslo, Norway; 22 Department of Pharmacy, University of Oslo, Oslo, Norway; 23 Child Health Research Centre, Level 6, Centre for Children’s Health Research, University of Queensland, 62 Graham Street, South Brisbane, QLD 4101 Australia; 24 Child and Youth Mental Health Service, Children’s Health Queensland Hospital and Health Service, Brisbane, Australia

**Keywords:** Childhood and adolescence psychopathology, Depression, Anxiety, Attention deficit hyperactivity disorder (ADHD), Psychiatric genetics

## Abstract

The Roadmap for Mental Health and Wellbeing Research in Europe (ROAMER) identified child and adolescent mental illness as a priority area for research. CAPICE (Childhood and Adolescence Psychopathology: unravelling the complex etiology by a large Interdisciplinary Collaboration in Europe) is a European Union (EU) funded training network aimed at investigating the causes of individual differences in common childhood and adolescent psychopathology, especially depression, anxiety, and attention deficit hyperactivity disorder. CAPICE brings together eight birth and childhood cohorts as well as other cohorts from the EArly Genetics and Life course Epidemiology (EAGLE) consortium, including twin cohorts, with unique longitudinal data on environmental exposures and mental health problems, and genetic data on participants. Here we describe the objectives, summarize the methodological approaches and initial results, and present the dissemination strategy of the CAPICE network. Besides identifying genetic and epigenetic variants associated with these phenotypes, analyses have been performed to shed light on the role of genetic factors and the interplay with the environment in influencing the persistence of symptoms across the lifespan. Data harmonization and building an advanced data catalogue are also part of the work plan. Findings will be disseminated to non-academic parties, in close collaboration with the Global Alliance of Mental Illness Advocacy Networks-Europe (GAMIAN-Europe).

## Introduction

The 2011 Roadmap for Mental Health and Wellbeing Research in Europe (ROAMER) [1–3] outlined six priorities for research in mental health, of which three are the focus of the EU funded Marie Skłodowska-Curie International Training Network CAPICE project: Childhood and Adolescence Psychopathology: unravelling the complex etiology by a large Interdisciplinary Collaboration in Europe:

1. Research into prevention, mental health promotion, and interventions in children, adolescents, and young adults;
2. Focus on the development and causal mechanisms of mental health symptoms, syndromes, and wellbeing across the lifespan (including older populations);
3. Develop and maintain international and interdisciplinary research networks and shared databases in the area of childhood and adolescence psychopathology.

To address these priorities, CAPICE brings together data from eight population-based birth and childhood cohorts, including twin cohorts, to focus on the causes of individual differences in childhood and adolescent psychopathology and its course (see Tables 1, 2). The CAPICE cohorts have collected longitudinal data on behavioral and emotional symptoms, lifestyle characteristics and environmental measures as well as genetic and epigenetic data (see Table 2), some also from parents. Data from other cohorts from the EArly Genetics and Life course Epidemiology (EAGLE) consortium [4], behaviour and cognition group, can also be involved in the studies, especially for genome-wide association meta-analysis (GWAMA). The EAGLE consortium is a collaboration of population-based birth, childhood and adolescent cohorts including the cohorts participating in CAPICE. These cohorts are mainly based in Western Europe and Australia as, to our knowledge, no comparable cohorts exist in other countries. If there are, they are very welcome to participate.

**Table 1:** General descriptions of the CAPICE cohorts

Cohort name	Descriptions
ALSPAC	The Avon Longitudinal Study of Parents and Children (ALSPAC) is a longitudinal pregnancy cohort which aimed to recruit all pregnant women in the former county of Avon with an expected due date between April 1991 and December 1992. Detailed information has continued to be collected on mothers, partners and children from 15,454 pregnancies in the cohort, this process has been described in detail elsewhere. Ethics approval for the study was obtained from the ALSPAC Ethics and Law Committee and the Local Research Ethics Committees. Informed consent for the use of data collected via questionnaires and clinics was obtained from participants following the recommendations of the ALSPAC Ethics and Law Committee at the time. Consent for biological samples has been collected in accordance with the Human Tissue Act (2004). Please note that the study website contains details of all the data that is available through a fully searchable data dictionary and variable search tool: http://www.bristol.ac.uk/alspac/researchers/our-data/
TEDS	The Twins Early Development Study (TEDS) is a longitudinal twin study that recruited over 16 000 twin pairs born between 1994 and 1996 in England and Wales through national birth records. More than 10 000 of these families are still involved in the study. TEDS is a representative sample of the population in England and Wales. Rich cognitive and behavioural data have been collected from the twins from infancy to emerging adulthood with data collection at ages 2, 3, 4, 7, 8, 9, 10, 12, 14, 16, 18, 19 and 21, enabling longitudinal genetically sensitive study designs. Data have been collected from twins themselves (including extensive web-based cognitive testing), from their parents and teachers, and from the UK National Pupil Database. More information may be found at: https://www.teds.ac.uk/. Ethical approval for TEDS has been provided by the King’s College London Ethics Committee (Reference number: PNM/09/10-104). Parental consent was obtained before data collection
GenR	The Generation R (Gen-R) study is a prospective cohort study from fetal life onwards that included pregnant women living in Rotterdam, the Netherlands, with an expected delivery date between April 2002 and January 2006 (*n* = 9778). The main aim of this study is to identify early environmental and genetic factors that affect growth, health and development. The Generation R Study is multidisciplinary and both prenatal and postnatal measures have included multiple domains of growth, health and development. Rotterdam is an ethnically diverse city and this is reflected in the Generation R participants. Of the enrolled mothers, 42% was of non-Dutch ethnic background, largely made by mothers from Surinamese (9%), Turkish (7%) and Moroccan (3%) background. Data has been collected in children up until the mean age of 10 years, with current on-going data collection at mean age 13 years. Study protocols were approved by the local ethics committee, and written informed consent and assent was obtained from all parents and children. More information may be found at: https://generationr.nl/researchers/
NTR	The Netherlands Twin Register (NTR) is a population-based prospective cohort study which includes new-born twins and multiples from the Netherlands. Recruitment started with birth year 1986. NTR data collection has a focus on growth, development, emotional and behavioural problems and health. Phenotype data on emotional and behavioural problems were collected by surveys, in which parents and teachers were asked to rate their offspring/pupils’ behaviour using standardized instruments. At age 14 years and after, twins and their siblings were asked for self-assessments. Buccal cells and blood for DNA isolation were collected in multiple sub-projects. More information may be found at: https://tweelingenregister.vu.nl/. The study was approved by the Central Ethics Committee on Research Involving Human Subjects of the VU University Medical Centre, Amsterdam, an Institutional Review Board certified by the U.S. Office of Human Research Protections (IRB number IRB00002991 under Federal-wide Assurance- FWA00017598; IRB/institute codes, NTR 03-180)
TCHAD	The Swedish Twin study of CHild and Adolescent Development (TCHAD) is a longitudinal study of how genes and environments contribute to the development of health and behavioural problems from childhood to adulthood. The study includes 1480 twin pairs followed since 1994, when the twins were 8–9 years old. The last data collection was in 2005 when the twins were 19–20 years old. Both parents and twins have provided data. More information may be found at: https://ki.se/en/meb/twin-study-of-child-and-adolescent-development-tchad. All responding parents, legal guardians or twins provided informed consent; digitally, written or by participation, to the study, and all data were deidentified. This study received ethical approval from the Karolinska Institutet Ethical Review Board
NFBC’86	Northern Finland Birth Cohort 1986 (NFBC1986) is a prospective longitudinal birth cohort which included pregnant women with expected date of delivery between July 1985 and June 1986 in the two Northern most provinces of Finland. In total, 9432 children were live-born in the cohort. At approximately age 16 years, the cohort members were asked to complete a postal questionnaire, including the Youth Self-Report (YSR). At age 16 years blood samples taken for DNA extraction for 6 266 adolescents attending the clinical examination. All participants and their parents provided consent to use their data and received Institutional Review Board approval by the University of Oulu, and the Ethics committee of the Ostrobotnia Hospital district. More information may be found at: https://www.oulu.fi/nfbc/
CATSS	The Child and Adolescent Twin Study in Sweden (CATSS) is an ongoing longitudinal twin study targeting all twins born in Sweden since July 1, 1992. Parents of twins are interviewed regarding the children’s somatic and mental health and social environment in connection with their 9th or 12th birthdays and followed up over time. Follow-ups were conducted when the twins were 15 years of age and 18 years of age. All responding parents, legal guardians or twins provided informed consent; digitally, written or by participation, to the study, and all data were deidentified. This study received ethical approval from the Karolinska Institutet Ethical Review Board. DNA samples (from saliva) were obtained from the participants at study enrolment. More information may be found at: https://ki.se/en/meb/the-child-and-adolescent-twin-study-in-sweden-catss
MOBA	The Norwegian Mother, Father and Child Cohort Study (MoBa) is a population-based pregnancy cohort study conducted by the Norwegian Institute of Public Health. Participants were recruited from all over Norway from 1999 to 2008. The women consented to participation in 41% of the pregnancies. The cohort now includes 114,500 children, 95,200 mothers and 75,200 fathers. Parent and infant DNA samples were collected at birth and stored in a biobank. The establishment of MoBa and initial data collection was based on a license from the Norwegian Data protection agency and approval from The Regional Committees for Medical and Health Research Ethics. The MoBa cohort is based on regulations based on the Norwegian Health Registry Act. The current study was approved by The Regional Committees for Medical and Health Research Ethics (REK 2013/863). More information may be found at: https://www.fhi.no/en/studies/moba/

**Table 2:** Summary of available data in the CAPICE cohorts**:** number of children with survey data and genome-wide association data (N GWA) and epigenetics data (N epi) at ages 3–5 years, 6–8 years, 9–11 years, 12–13 years, 14–15 years, and 16–18 years

Cohort	N GWA	N epi	3–5	6–8	9–11	12–13	14–15	16–18
ALSPAC	8237	1018	S	S	S	S		S
TEDS*	10,346		S	S	S	S		S
GenR	2211	1500	A	A	A	A		
NTR*	6400	1400	A	A	A	A	A	A
TCHAD*	1120			A		A		A
NFBC’86	4000	530		S				A
CATSS*	11,400				O**	O**	S	
MOBA	90,000		A	O***				
Overall total	73,714	4448						

The first rows summarize the cohorts that assessed mental health symptoms either with the strengths and difficulties questionnaire (S) or the Achenbach system of empirically based assessment (A), the following row the cohorts with S and A measures, and the final rows the cohorts with other (O) measures

^*^Number of twins. O** A-TAC: Autism–Tics, AD/HD and other Comorbidities inventory, O***SCARED: screen for child anxiety related emotional disorders, MFQ: mood and feelings questionnaire

**Fig. 1:**
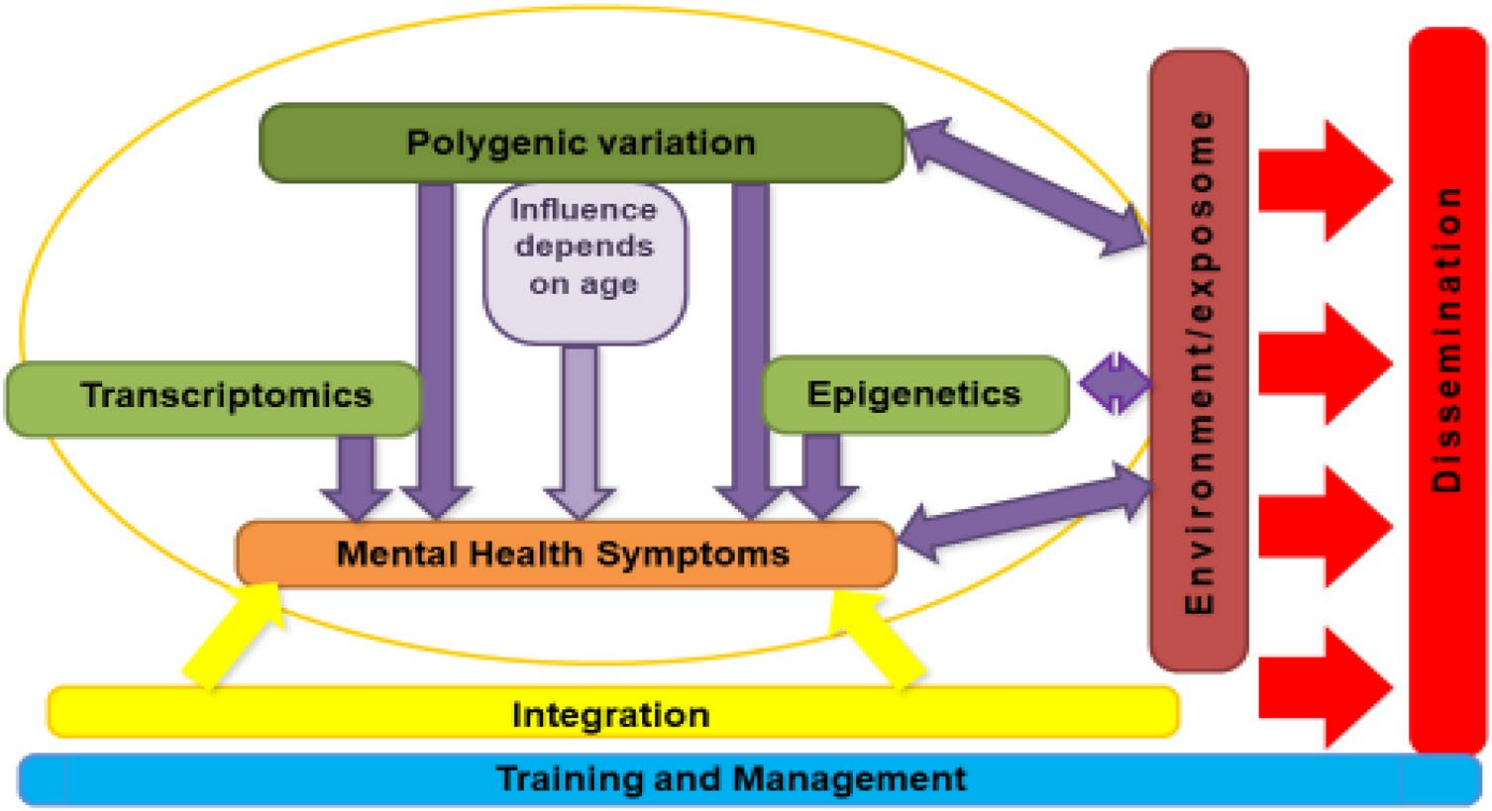
Overview of the CAPICE research programme: the aims are to investigate the influence of genetic, epigenetic, and transcriptomic variants on mental health symptoms, the interplay with the environment, and how these influences depend on age. Ultimately, these results will be integrated into a model predicting the persistence of symptoms

Efforts to prevent and treat childhood psychopathology need to be informed by a clear understanding of the aetiology of mental disorders, and factors that impact the development of a chronic course. Due to the genetically informative, longitudinal designs of the eight included cohorts, CAPICE is well positioned to address questions regarding the interplay of genetic and environmental factors in the development, course, and comorbidity patterns of child psychiatric conditions. Since CAPICE is an international training network, the analyses have been performed by 12 early stage researchers under the supervision of senior researchers at academic and non-academic sites across 5 European countries (Italy, The Netherlands, Norway, Sweden, and the United Kingdom). In this paper, we describe the six CAPICE objectives, as well as the methodological approaches that have been used and the initial results. The overview of the CAPICE research programme is illustrated in Fig. 1.

## Objectives

1. Elucidate the role of genetic and environmental factors in mental health symptoms across childhood and adolescence, and to establish the overlap in genetic risk factors with other traits related to childhood mental health symptoms.
  The projects that are part of this objective focus on the investigation of the overlap in genetic risk across phenotypes and ages, to explain comorbidity and persistence, respectively. Moreover, analyses will focus on disentangling the role of genetic and environmental factors in associations between childhood mental health symptoms and parental phenotypes or risk factors early in life.
  Previous research suggests that both genetic and non-genetic factors play a role in the development and persistence of mental health symptoms and disorders. Depending on age, gender, and type of mental health symptoms, genetic factors explain between 40 and 80% of their symptom variance between individuals [5], indicating that the contributions of genetic and environmental factors vary across disorders. Identifying mechanisms that underlie the persistence of symptoms and patterns of comorbidity are of specific importance in childhood psychopathology due to the potential impact of these disorders throughout the life course. Epidemiological studies have shown that about 50% of children with mental disorders still suffer from mental disorders in adulthood [6], including severe mental illnesses such as schizophrenia (SCZ) [7]. As comorbidity has been associated with worse prognosis [6, 8], it is essential to comprehend the mechanisms behind this.
  It is also still unknown to what extent the associations between environmental factors and mental disorders are explained by causal processes, in which the environmental risk factor directly increases the risk for psychopathology, or by other processes such as, for example, gene–environment correlation, where the same genetic variants that influence a child’s risk for a mental disorder are associated with the child’s risk to be exposed to an unfavourable environment.
2. Identify genetic, epigenetic, and transcriptomic variants associated with mental health symptoms during childhood and adolescence.
  There has been considerable progress in the field of psychiatric genetics with over two hundred genomic regions significantly associated with SCZ [9], bipolar disorder (BD) [10], depression [11, 12], attention-deficit/hyperactivity disorder (ADHD) [13], and autism spectrum disorder (ASD) [14]. This has been achieved by large international collaborations such as the Psychiatric Genomics Consortium (PGC), performing meta-analyses with total sample sizes even up to 100,000 subjects [12]. The number of variants which have been associated with childhood psychopathology at genome-wide significance is still lower than for adult mental disorders [13, 14], but polygenic analyses have suggested that genetic variation in childhood mental disorders, as in adult disorders, is primarily due to many genetic variants of small effect [15]. This would mean that genome-wide association studies (GWAS) of child and adolescent psychopathology, which so far have been notably smaller than the large meta-analyses above, have likely been underpowered. By increasing the sample size and performing meta-analyses across the EAGLE cohorts, including the CAPICE cohorts, it may be possible to detect genome-wide significant associations.
  In addition, because extended longitudinal data on mental health problems are available, it is possible to test whether genetic variants exert their influence across the lifespan or if their effect is restricted to a certain age period.
  Finally, as previous work has also suggested that the impact of environmental factors on childhood psychopathology may be mediated by epigenetic variation (functional alterations in the genome that do not involve a change to DNA sequence [16]), identification of epigenetic differences associated to childhood mental disorders is also part of this objective.
3. Identify biological pathways associated with mental health symptoms and to validate potential drug targets based on these pathways.
  The current identified genetic variants (SNPs) for psychiatric disorders only explain a small part of their variance [17–19]. However, the joint effect of all tested variants explains on average around 30% of the variance of psychiatric disorders [20], which makes it worth to investigate these variants further [21]. A key step in the pathway from variant identification in GWAS to the development of potential treatment or prevention approaches is the characterization of biologic pathways by which genetic variants affect disorders. For instance, follow-up analyses can specify whether associated variants are involved in the same biological pathways, which then can offer leads to novel drug targets or the repurposing of existing drugs that were initially developed for the treatment of other diseases [21–23].
  In a recent study [22], a framework for drug repositioning is offered by associating transcriptomes imputed from GWAS data with drug-induced gene expression profiles from the Connectivity Map database. When applied to mental disorders, the method identified many candidates for repositioning, several upheld by preclinical or clinical evidence as they included known psychiatric medications or therapies considered in clinical trials [22]. Validation studies of these approaches have recognized antipsychotics as potential drug targets for mental disorders, confirming the approach may be a productive technique for developing drug therapies for childhood psychiatric conditions [24].
4. Build a prediction model that identifies children at the highest risk of developing chronic mental health symptoms.
  To be able to provide treatment that takes into account the risk for persistence of symptoms, it is necessary to build a prediction model that supports stratifying children into groups at high and low risk for a severe, chronic symptom course. Similar risk calculators based on clinical symptoms and cognitive profiles have already been established for high-risk individuals for SCZ or BD [25–27]. Including environmental factors and multi-omic biomarkers may further improve the performance of the model [28].
5. Develop a sustainable international network of researchers in which collaboration is facilitated by data harmonization and information technology (IT) solutions enabling a joint analysis of data over cohorts.
  The eight cohorts involved in CAPICE contain a wealth of data on childhood and adolescent mental health, but measures used to assess these traits differ across cohorts (see Table 2). One way to improve the power of studies combining results from multiple cohorts is to use common units of measurement. Using item-response theory (IRT) based test linking in these cohorts; it is possible to evaluate the extent to which dimensions from the individual instruments can be mapped onto dimensions that are shared across instruments. Such an analysis was successfully applied earlier to measures of personality [29].
6. Build a structure to disseminate the results to a broad audience of scientists, clinicians, patients and their parents, and the general public.
  A limitation of research projects consists in the difficulty of properly disseminating their results, even if clinically relevant. Previous work has suggested that, even in targeted clinical research, it takes 17 years for 14% of discovery research to be integrated into physician practice [30]. Etiological research on mental disorders may include even broader dissemination gaps. To address this issue, CAPICE specifically aims to create structures allowing for the dissemination of project results to a broad audience, for instance, through a website and social network channels.

## Methodology and results

### Cohorts description

Before describing the approaches and results of each objective, we provide a general summary of the data collection of the eight CAPICE cohorts (see Tables 1, 2). All longitudinal cohorts have started either at birth or in childhood and use quantitative measures of psychopathology. These measures have been associated with clinical diagnoses [31–34] and are therefore widely used in clinical practice. The advantage of these dimensional measures is that they capture more of the variation present in the common population than dichotomous measures that only specify the presence or absence of a diagnosis. For genome-wide SNP data, methods to impute the genotypes are widely available allowing for the same genetic variants to be analysed over cohorts. All cohorts have been described in more detail elsewhere: ALSPAC [35–37], TEDS [38], GenR [39, 40], NTR [41–43], TCHAD [44], NFBC’86 [45], CATSS [46, 47], MOBA [48]. For a link to cohort-specific websites and for a detailed description of the cohorts (see Table 1). All data used for the analyses were collected under protocols that have been approved by the appropriate ethics committees, and studies were performed in accordance with the ethical standards.

Objective 1: Elucidate the role of genetic and environmental factors in mental health symptoms across childhood and adolescence, and to establish the overlap in genetic risk factors with other traits related to childhood mental health symptoms.

We refer to several excellent overviews for a description of the methods that can be used in genetic epidemiological studies analysing twin/family data and/or molecular genetic data [15, 49, 50]**.** In short, twin and family studies estimate the proportion of variance in a trait attributable to genetic and environmental factors by comparing the resemblance between pairs of relatives that differ in their relatedness. For example, if monozygotic twins, who are essentially 100% genetically identical, are more alike than dizygotic twins, who share on average 50% of their co-segregating alleles, this is an indication that genetic factors play a role in explaining differences between individuals for a certain trait. This model can be extended to include other family members, to longitudinal or multivariate designs, and to study gene–environment interaction (G × E), even without a direct measure of the environment [51–53].

It is also possible to address these questions using genotypic data obtained for GWAS. In GWAS, common single nucleotide polymorphisms (SNPs) (positions in the DNA sequence that vary between individuals) measured across the whole genome are tested for their association with a trait. Many complex traits, like mental disorders, are influenced by multiple genetic loci. In this case, polygenic analyses, taking into account many SNPs, can be applied to investigate the cumulative impact of these SNPs on a trait, as well as on the co-occurrence of phenotypes or the persistence of symptoms over time [15, 49]. To this point, CAPICE researchers have performed twin and polygenic risk score analyses of the correlation between common mental health symptoms, such as internalizing problems and ADHD problems, and the stability of symptoms over time, including into adulthood.

Regarding the co-occurrence of symptoms, the covariance could be explained by one common factor, the so-called *p*-factor, which was found to be for 50–60% heritable. Moreover, genetic factors explained stability in this factor across ages. A polygenic *p*-factor risk score based on adult psychiatric disorders was also associated with the childhood *p*-factor [54].

The genetic association between adult psychiatric disorders and childhood and adolescents traits was further investigated with polygenic analyses. The PGS for adult depression, neuroticism, BMI, and insomnia were significantly positively associated with childhood ADHD, internalizing and social problems, while the PGS for subjective well-being and educational attainment showed negative associations [55]. Only bipolar disorder PGS did not yield any significant associations. Effect sizes were in general similar across age and phenotype, although the PGS for educational attainment was more strongly associated with ADHD and the BMI PGS with ADHD and social problems [55]. A follow-up study is currently on the way, performing multivariate analyses to shed more light on the pattern of associations (https://osf.io/7nkw8).

Genetic data can also be leveraged to estimate the average causal effect of specific environmental factors, such as perinatal factors, on child or adolescent psychopathology, using a method known as Mendelian randomization (MR). Several ongoing CAPICE projects have applied this approach to estimate the effect of prenatal risk factors, such as maternal smoking, on childhood mental health problems. It is important to recognize that the assumptions that are required for MR may not hold for all prenatal exposures and offspring outcomes [56]. Therefore, methods to identify violations of the MR assumptions are also evaluated and approaches that require fewer assumptions are tested.

Another method to analyse the mechanisms underlying parent-offspring associations is maternal genome-wide complex trait analysis (M-GCTA), which can be applied when parental genotypic data are also available. M-GCTA can be used to calculate whether the association between parent and offspring psychopathology is explained by an environmental effect on top of the effect of the genetic transmission [57]. Applying this method to the MoBa data indicated no such effects for anxiety and depression at age 8 [58]. Analyses on a larger so more powerful sample and including externalizing problems are currently performed.

Objective 2: Identify genetic, epigenetic, and transcriptomic variants associated with mental health symptoms during childhood and adolescence.

GWAS have provided insight into the genetic basis of quantitative variation in complex traits in the past decade [20]. By increasing the sample size and performing meta-analyses across the EAGLE cohorts, including the CAPICE cohorts, it may be possible to detect genome-wide significant associations and to detect age effects. A large-scale GWAMA using a multivariate method to analyse summary statistics that are not independent [59] focused on identifying genetic variants that influence the development and course of internalising symptoms from ages 3 to 18 (https://osf.io/w5adg/). Three gene-wide significant effects were detected as well as significant genetic associations with adult depression and related traits as well as with childhood traits. Another study explores the effect of (ultra) rare and common variation in genes specific to brain cell types on neuropsychiatric disorders (https://osf.io/uyv2s).

Previous work has also suggested that the impact of environmental factors on childhood psychopathology may be mediated by epigenetic variation, which consists of functional alterations in the genome that do not involve a change to DNA sequence [16]**.** While there are several forms of epigenetic variation, most epigenetic studies have focused on alterations in DNA methylation. Epigenome-wide association studies (EWAS) are performed to test the effect of maternal mid-pregnancy vitamin D on offspring cord blood methylation and of the association between variation in child peripheral and cord blood methylation and the subsequent development of ADHD.

Under very strong assumptions, mediation of the possible average causal effect of prenatal exposures on offspring psychiatric outcomes might be tested using an extension of the MR approach, incorporating genetic variants as proposed instruments for a particular prenatal exposure and for methylation at a specific locus. In certain contexts, this might be considered as a follow up to the present studies.

The association of prenatal maternal smoking with offspring blood DNA methylation has been investigated in individuals aged 16–48 years, and MR and mediation analyses have been performed to evaluate whether methylation markers have causal effects on disease outcomes in the offspring [60]. 69 differentially methylated CpGs in 36 genomic regions (*P*-value < 1 × 10^−7^) were found to be associated with exposure to maternal smoking in adolescents and adults and MR analyses delivered evidence for a causal role of four maternal smoking-related CpG sites on an increased risk of SCZ or inflammatory bowel disease [60]. Further studies analyse whether alcohol, tobacco, and caffeine use in pregnancy might be causally related to ADHD in the offspring using negative control and MR approaches (https://osf.io/wxu58) (https://osf.io/aqrxp).

Objective 3: Identify biological pathways associated with mental health symptoms and to validate potential drug targets based on these pathways.

Applying drug pathway analyses to the CAPICE GWAS results may permit us to derive hypotheses about potential drug targets and consequently possibilities for drug repurposing. The GWAMA on internalizing problems did not detect biological pathways (https://osf.io/w5adg/) so could not identify drug targets. This is not surprising as there were not many significantly associated genes.

Objective 4: Build a prediction model that identifies children at the highest risk of developing chronic mental health symptoms.

Using cohort data, as well as Swedish registry data, studies have been performed to predict outcomes of psychiatric symptoms in childhood and adolescence, focusing not only on mental disorders but also on somatic medical outcomes. As part of these analyses, a machine learning model including 474 predictors has been developed that can predict mental health problems in adolescence using data from the Child and Adolescent Twin Study in Sweden (CATSS) [61]. The suggested model would not be appropriate for medical purposes, but it helps to build better models to predict mental health outcomes [61].

Moreover, longitudinal analyses of data from the Swedish and Dutch twin registers indicated that adolescent anxiety is associated with psychiatric disorders later in life, even when adjusting for other mental health issues [62].

Objective 5: Develop a sustainable international network of researchers in which collaboration is facilitated by data harmonization and information technology (IT) solutions enabling a joint analysis of data over cohorts.

Using Item-Response Theory (IRT) based test linking it has been evaluated whether internalizing and ADHD symptoms assessed by different instruments can be mapped onto dimensions that are shared across instruments. These analyses were possible as some of the EAGLE cohorts (ABCD [63], Raine [64], and TEDS [38]), had measured mental health symptoms of the same individuals at the same age with two or more instruments. This could allow combining individual raw item data from different instruments to maximize statistical power.

In addition, to facilitate data analyses over cohorts, a searchable data catalogue is created. The variables important for the current project include demographic and family characteristics, individual’s school achievements, mental health measures (both psychopathology as well as wellbeing) by various raters (mother, father, self-report, teacher), pregnancy/perinatal measures, several general health and anthropometric measures, parenting, parental mental health, and several genomic measures and biomarkers in children and parents. To build a search engine that returns items including the searched term as well as related terms, text mining of available data documentation has been used to identify relationships between words. These results can then be used to develop an advanced search engine for the data catalogue. If “mental health” is, for example, the search term, the results will also include “emotional problems”, “behavioural problems”, and “psychiatric history of the mother”.

Objective 6: Build a structure to disseminate the results to a broad audience of scientists, clinicians, patients and their parents, and the general public.

To engage the general public with the results from these studies, CAPICE researchers have also created content designed for a lay audience on the website (http://www.capice-project.eu/index), Twitter (https://twitter.com/capice_project), YouTube (https://www.youtube.com/channel/UCgq8uIHiHE69IlcHoYCjwKg/featured?view_as=subscriber), LinkedIn and Facebook. CAPICE was also represented at the Greenman Festival in Wales, UK, and ESRs presented multiple times on several international conferences.

## Discussion

Genetic research, including psychiatric genetics, has substantially moved forward due to large-scale collaborations in consortia, with meta-analyses being the rule rather than the exception. Due to developments in methodology, it is also possible to use the genome-wide genotypic data for purposes other than the identification of genetic risk variants. Given the small effect sizes, these analyses need large samples to achieve adequate statistical power. The cohorts brought together in CAPICE and the close collaboration with the EAGLE behavior and cognition group (https://www.eagle-consortium.org/) has provided an opportunity to perform these analyses and progress the field of child psychiatry by addressing essential questions like “Which genetic variants and biological pathways underlie the continuity of symptoms from childhood into adulthood?”; “Which factors explain the associations of childhood psychopathology with early life and familial risk factors?”; “What is the role of epigenetic factors in the development of the child and adolescent psychopathology?”; “Can we predict which children are at higher risk for poorer outcomes?”.

The ultimate aim is that these results will inform the development of future treatment and prevention efforts, including supporting the identification of novel targets for existing pharmacological agents. Moreover, having better prediction tools will bring precision medicine closer for child psychiatry as it provides the opportunity to test interventions specifically targeted at children at high or at low risk for the persistence of symptoms.

We acknowledge that the studies performed in the framework of CAPICE are largely restricted to children and families with a Western European genetic ancestry. Extending genetic data collection to children and families from other backgrounds is essential to gain knowledge on similarities and differences between groups from various backgrounds.

These analyses have all been performed as part of a training program for Early Stage Researchers. These researchers receive not only mentoring from senior academics on their specific projects but also a structured curriculum of workshops on child psychiatry, statistics, and dissemination strategy throughout the grant period. These workshops as well as the secondments at other participating institutions aim to build a new generation of creative and innovative researchers who might exert a relevant impact on academic and non-academic organizations.

In conclusion, CAPICE provides a broad package of training in the field of psychiatric genetics, going from harmonizing the phenotypes, the creation of facilities for analyses across cohorts and the actual state-of-the-art analyses, to the translation of the results for drug target validation or prediction models that can be used in the clinic for targeted interventions.
